# Characteristics of Community-Dwelling Older People Who Are Less Likely to Respond to Mail Surveys Under Infection Countermeasures for New Strains of Coronavirus: The Takasaki Study

**DOI:** 10.3390/ijerph22030437

**Published:** 2025-03-16

**Authors:** Akihiko Murayama, Daisuke Higuchi, Kosuke Saida, Shigeya Tanaka, Tomoyuki Shinohara

**Affiliations:** 1 Department of Physical Therapy, Faculty of Rehabilitation, Gunma University of Health and Welfare, Maebashi Plaza Genki 21 6-7F, 2-12-1 Hon-machi, Maebashi-shi 371-0023, Gunma, Japan; 2 Department of Physical Therapy, Faculty of Health Care, Takasaki University of Health and Welfare, 27 Naka Orui-machi, Takasaki-shi 370-0033, Gunma, Japan; higuchi-d@takasaki-u.ac.jp (D.H.); saida@takasaki-u.ac.jp (K.S.); tanaka-s@takasaki-u.ac.jp (S.T.); shinohara-t@takasaki-u.ac.jp (T.S.)

**Keywords:** Takasaki study, community-dwelling older people, mail surveys, face-to-face support, community care networks

## Abstract

This study aimed to identify the characteristics of community-dwelling older people who are difficult to reach by mail survey in anticipation of a future infectious disease crisis. A baseline survey of 1808 community-dwelling older people was conducted in May 2021, and a follow-up survey of 935 respondents was conducted in May 2023. Factors predictive of responding to the follow-up survey included age at baseline, sex, comorbidities, living with family, long-term care insurance, a history of falls, the Simple Frailty Index, and a Questionnaire on Changes in Lifestyle in the Past Month (QCL). Participants were divided into the responding (n = 330) and non-responding (n = 605) groups. Binomial logistic regression analysis was used to analyze items that showed significant differences in the between-group comparison: odds ratios (ORs) of 2.36, 1.84, 1.69, 1.57, and 1.20 for living alone, having comorbidities, having long-term care insurance, fatigue, and reduced ability to communicate, respectively. If social distancing is required in the future, we believe that face-to-face support should be prioritized for people who live alone, have comorbidities, use long-term care insurance, or are aware of fatigue and limited communication, as it is highly unlikely that they will be able to continue exchanging written information.

## 1. Introduction

Japan uses the term “older people” to denote individuals aged ≥ 65 years. On 17 September 2024, the population of older people in Japan reached a record high of 36.25 million, comprising 29.3% of the total population. Japan has the highest proportion of older people worldwide, as confirmed by data from 200 countries and regions [1]. Japan’s “healthy life expectancy”, defined as the predicted length of time that Japanese people can expect to maintain good health, has been calculated to be 75.45 years for females and 72.57 years for males in 2022. A comparison with a survey conducted 3 years prior by the Ministry of Health, Labor, and Welfare revealed that the healthy life expectancy of older individuals has been increasing, although the spread of coronavirus disease 2019 (COVID-19) has led to the hypothesis that this trend may have been halted [2].

It has been suggested that pandemic recovery efforts should prioritize the most affected groups to reduce health disparities and ensure equitable recovery [3]. During the COVID-19 pandemic, delays in medical care were widespread. This delay in necessary care for older people beyond medical services posed a significant challenge. For this reason, ensuring that vulnerable populations, mainly older people, receive essential care even during a pandemic has been emphasized [4]. Previous studies have shown that older people with poor self-rated health were more likely to experience delays in care during the COVID-19 pandemic [5,6,7]. Such delays may exacerbate short- and long-term changes in their health status. On the other hand, older people who are more prone to delayed medical care may benefit from targeted follow-up [8]. This suggests the importance of understanding the adverse health effects of care delays in older people, which can offer valuable insights into the long-term health needs of the post-pandemic population [9].

In particular, the incidence of frailty was twice as high among older people who lived alone and had lower social activity than among those who did not live alone or had high social activity [10]. Therefore, it can be concluded that the pandemic has had a deleterious effect on physical activity levels in older people residing in Japan [11]. Furthermore, an increased use of infection prevention measures was associated with a greater negative impact on quality of life and social participation, which was more pronounced among older people with complex health problems [12]. Older people with comorbidities face significant challenges, especially during the pandemic, because comorbidities affect them at many levels: physiologically, functionally, psychologically, and socially [13,14]. In fact, following the COVID-19 outbreak, the psychosocial health of older people with multimorbidities worsened significantly, and missed medical appointments increased significantly [15,16,17]. While addressing this unknown infectious disease is urgent, we must also consider the health status of older individuals who face unique challenges. However, information on older people during the pandemic remains limited [18].

Shinohara [19,20] conducted a health survey during the spread of COVID-19 in Takasaki City, Gunma Prefecture, from 2020 to 2021. This valuable cohort study was conducted during the early stages of the COVID-19 spread in Japan. Hereafter, we refer to this as the Takasaki Study. The vision of the Takasaki Study is to take measures to effectively prevent frailty by screening older people who are expected to need a high level of support and by taking measures that focus on the specific aspects of daily life that should be considered. In other sub-analyses, we examined items related to subjective cognitive decline (SCD) [21]. We examined fall predictors during the COVID-19 pandemic [22,23]. In the Takasaki Study, a follow-up survey was conducted in May 2023 to capture the actual situation 3 years after the spread of COVID-19. This study is the result of a sub-analysis using data from the Takasaki Survey, focusing on the characteristics of community-dwelling older people for whom it is difficult to obtain responses to mail surveys during the spread of COVID-19. In anticipation of a future infectious disease crisis, this study aimed to clarify the characteristics of community-dwelling older people for whom it is difficult to obtain responses to mail surveys. In addition, this study focused on whether characteristics such as living alone and comorbid conditions, which were identified as risk factors for frailty in community-dwelling older people during the COVID-19 outbreak, were consistent with the characteristics of community-dwelling older people for whom mail surveys were difficult to conduct. We believe that understanding the longitudinal relationship between such behavioral changes due to the COVID-19 pandemic and health outcomes is important when considering health promotion approaches in the post-COVID era.

## 2. Materials and Methods

### 2.1. Study Design and Participants

This prospective cohort study included community-dwelling older people aged ≥ 65 years who resided in Takasaki City. Of the 35 municipalities in Gunma Prefecture, Takasaki City is distinguished by its status as the most populous. According to the Basic Resident Register, Takasaki City has a total population of 368,196, with 105,696 individuals categorized as older, representing an older population percentage of 28.71%. The proportion of single-person households among older households was 28.6%. The nursing care certification rate, a metric indicative of the utilization of nursing care insurance, was 17.4%. However, the survey is inexhaustive.

**Figure 1:**
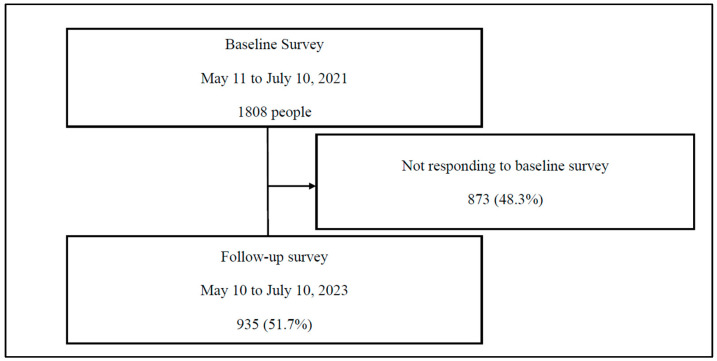
Flowchart of the participant selection process.

The preliminary phase of the study involved the administration of a baseline survey of 1808 community-dwelling older people. The study population consisted of 935 individuals identified by tracing from the initial baseline survey to subsequent follow-up surveys (Figure 1). The baseline survey was conducted from 11 May 2021 to 10 July 2021. At the time of the baseline survey, the number of new daily viral infections in Gunma Prefecture was 84.7, the highest ever recorded. Concurrently, the Governor of Gunma Prefecture formally requested the implementation of priority measures by the national government to prevent the spread of the virus [24]. Subsequent to the baseline survey, a follow-up survey was conducted from 10 May to 10 July 2023, to ascertain the actual situation after a two-year interval. The subsequent survey was administered when the Japanese government declared its intention to transition from a pandemic management system characterized by the issuance of various requests and involvement in accordance with legal provisions to a response that acknowledges individual autonomy and is founded on the voluntary actions of the public [25].

The civil welfare commissioners were assigned the task of conducting regular home visits to study the participants, in addition to distributing questionnaires and research instructions to prospective participants. Individuals who expressed interest in participating in the study were requested to return questionnaires and consent forms for research participation by postal mail; the survey materials were distributed by mail. The participants were contacted by telephone to provide explanations and confirm the safety of the study. The use of postal mail for the return of questionnaires was a strategic decision that allowed research staff to efficiently identify participants who had completed and returned the forms and to compile the necessary data.

This study was conducted in accordance with the Declaration of Helsinki and the ethical guidelines for medical science research involving human subjects. This study was approved by the Research Ethics Committee of Takasaki University of Health and Welfare (approval numbers 2009 and 2259) and registered with the University Hospital Medical Information Network (UMIN000040335). Before performing the study, all participants received a comprehensive questionnaire outlining the study objectives, scope, and contact information for inquiries. Participants were required to provide their names and formal consent before inclusion in the study.

### 2.2. Measurements

To predict whether the participants would respond to the mail survey in the subsequent follow-up survey, a comprehensive set of variables was investigated. These variables encompassed baseline age, sex, comorbidity, living arrangement, long-term care insurance, a history of falls in the past 6 months (“fall history”), the Simple Frailty Index (“FSI”) total score and sub-items [26], and the Questionnaire on Changes in Lifestyle in the Past Month (“QCL”) [27]. Furthermore, the probability that participants would respond to the mail survey in the subsequent follow-up survey was assessed (henceforth, “outcome”).

The FSI was assessed using a self-administered questionnaire consisting of five binary (yes/no) questions. A score of 3 or higher indicated the presence of frailty, while scores between 1 and 2 suggested prefrailty. The FSI was carefully developed to systematically track changes in frailty among older individuals. In particular, the FSI showed a high degree of accuracy in identifying frailty in older Japanese individuals [28]. The Japanese version of the Cardiovascular Health Study criteria [29] is used to evaluate frailty in Japan. This encompasses the evaluation of ambulatory velocity and manual dexterity. This study employed the FSI, an alternative approach that utilizes questionnaire-based methods and does not require the collection of empirical measurements.

The QCL scale consists of five items designed to minimize response burden, thereby enabling older adults to complete the survey independently. This study incorporated a diverse range of variables, including activity levels associated with physical frailty and lower-extremity strength [30], dietary intake [31], activity levels linked to mental and psychological frailty and anxiety [32], and opportunities for interaction associated with social frailty [33]. The investigation employed a five-point scale to assess responses, facilitating a comprehensive quantitative analysis of participants’ self-reported health status. To evaluate the impact of measures implemented to prevent the spread of infection on lifestyle and physical and mental status, the subjective changes experienced over the period of social change caused by the ongoing pandemic and during the previous month were assessed. The data were subjected to a quantitative analysis, in which each item was assigned a score ranging from 1 to 5. A score of 1 indicated an increase or strengthening, a score of 2 indicated a slight increase or strengthening, a score of 3 indicated no change, a score of 4 indicated a slight decrease or weakening, and a score of 5 indicated a decrease or weakening. The worry and anxiety items are scored from 1 (decreased) to 5 (increased). The validity of most QCL items related to frailty has been demonstrated in previous studies [34].

Baseline age, sex, comorbidity, living with family, long-term care insurance, fall history, QCL items, FSI scores, and sub-items were compared between those who responded by mail at follow-up (“responding group”) and those who did not (“non-responding group”).

### 2.3. Statistical Analyses

To determine the sample size required for interval estimation of population proportions, statistical principles were applied based on an established methodology. The margin of error was set at 5% with a confidence level of 90%. The inherent challenge of predicting the composition of a population prompted the proposal of a predicted value of 50%. As a result, the final sample size was determined to be 385, and the R package naniar [35] was employed to analyze the missing data. In instances where the exclusion of missing values resulted in data bias, the R package mice was used to impute missing values. This multiple-assignment method is primarily oriented toward the completion of missing values in multivariate datasets. We used the R package naniar [35] to check for any bias introduced by excluding missing values from the dataset. The R package naniar [35] was used to determine whether the exclusion of missing values introduced bias into the dataset. Accordingly, missing values were subsequently imputed using the multiple imputation method (R package mice [36,37]), which requires two parameters: m for pseudo-complete datasets and the imputation method. In this study, the value of m was set to 10, and Predictive Mean Matching was used, which randomly chose and replaced observations that were close to the regression values. The results are summarized using Rubin’s rule [38]. The normality of the data was assessed using the Shapiro–Wilk test. Data from the responding and non-responding groups were normally distributed. We conducted unpaired *t*-tests to compare the means of age, FSI score, and individual QCL items between independent groups. Chi-square tests were used to test for differences in sex, comorbidities, living with family, long-term care insurance, a history of falls, and the proportion of FSI sub-items. Fisher’s exact test was used when the expected value was <5. Binary logistic regression analysis was used to calculate odds ratios (ORs) and 95% confidence intervals (CIs). The presence or absence of a response to the follow-up survey was used as the dependent variable and items with significant differences were used as independent variables. The forced entry method was used as an independent variable. In the forced entry method, variance inflation factors were assessed and items without multicollinearity were selected. Multivariate analysis was used to identify independent predictors. Previous studies have emphasized the “one in ten” rule for each predictor variable [39]. Each predictor variable included at least 10 participants. If this rule of thumb was not satisfied, the region was automatically classified as having a high risk of bias. Statistical analyses were performed using EZR software [40] and R Commander version 1.68 at a 5% significance level.

## 3. Results

The responding (n = 330) and non-responding (n = 605) groups were analyzed. Comparisons between the groups showed significant differences in sex (*p* < 0.05), living arrangement (*p* < 0.001), comorbidity (*p* < 0.001), long-term care insurance (*p* < 0.05), FSI score (Table 1), FSI sub-items (Q5, *p* < 0.05) (Table 2), and QCL (Q5, *p* < 0.05) (Table 3).

Binary logistic regression analysis was performed with the outcome as the dependent variable and the six items with significant differences (excluding the FSI score) as explanatory variables (adjusted for age and a history of falls). As a result, the following associations were extracted: living alone (OR 2.36, 95% CI: 1.71–3.27, *p* < 0.001), comorbidity (OR 1.84, 95% CI: 1.38–2.47, *p* < 0.001), having long-term care insurance (OR 1.69, 95% CI: 1.15–2.49, *p* < 0.01), FSI sub-item (Q5) (OR 1.57, 95% CI: 1.10–2.23, *p* < 0.05), and QCL (Q5) (OR 1.20, 95% CI: 1.02–1.42, *p* < 0.05). The model χ^2^ test result was *p* < 0.001, and the Hosmer–Lemeshow test result was *p* = 0.931, indicating a good fit (Table 4).

## 4. Discussion

In this study, we focused on factors such as living alone and comorbidities, which increased the risk of progression to frailty during social distancing during the COVID-19 pandemic, and differences in the characteristics of community-dwelling older people who are difficult to survey by mail. The responding (n = 330) and non-responding (n = 605) groups were analyzed. Significant differences were observed between the two groups in the following aspects: living with family (77.6% vs. 60.5%) and female participants (78.5% vs. 72.4%). The responding group had a lower prevalence of comorbidities (56.4% vs. 70.2%), use of long-term care insurance (16.4% vs. 22.0%), and reported fatigue (19.7% vs. 26.3%). The mean FSI score was lower in the responding group (1.19 vs. 1.38), as was the QCL score for reduced ability to communicate (2.18 vs. 2.32).

Based on these results, a binary logistic regression analysis was performed, adjusting for age and a history of falls. The analysis identified the following associations: OR 2.36 for living alone, OR 1.84 for having a comorbid condition, OR 1.69 for having long-term care insurance, OR 1.57 for fatigue, and OR 1.20 for reduced ability to communicate. These findings suggest that individuals who live alone and those with comorbidities may face greater challenges in mail surveys. In addition, it is necessary to focus on characteristics such as the use of long-term care insurance, recent experiences of fatigue, and the frequency of communication with others.

In May 2023, the Japanese government reclassified COVID-19 as a Class 5 infectious disease, marking a shift in the nation’s infectious disease management system. This shift involved a transition from a system of legal requirements and government involvement to a response based on voluntary efforts, while respecting individual choices [25]. However, it is likely that despite this reclassification, many individuals experience residual discomfort regarding a complete return to their previous lifestyles [41,42]. It should also be noted that the World Health Organization [43] has highlighted the need to accumulate knowledge, including the prediction of pathogens (disease X) that can cause future epidemics or pandemics. Based on these considerations, we proposed a reevaluation of the results in the context of the following points.

Based on our findings, we believe that if social distancing is needed again in the future, it is likely that community-dwelling older people who live alone, have comorbidities, use long-term care insurance, and are aware of fatigue and reduced communication opportunities will need to be prioritized for face-to-face support, as they are unlikely to be able to continue to exchange information by mail. Even when providing face-to-face support, it may be effective to take care to maintain opportunities for communication with residents who meet the basic attributes of living alone, have comorbidities, and have long-term care insurance by asking about their recent feelings of fatigue. It is hoped that mail surveys conducted in situations where social distancing is required will not only benefit older people who respond but also reduce the disadvantages for older people who do not (or cannot) respond.

It is necessary to shift from a one-sided perspective on the health vulnerability of older people during the COVID-19 pandemic to accumulate knowledge about the multifaceted COVID-19 experience; however, relevant research is limited [44,45]. To date, no clear recommendations have been made to address these issues in Japan. One reason for this may be that community-dwelling older people have more diverse environmental factors than those who are hospitalized or institutionalized. The COVID-19 pandemic has highlighted a structural issue: a comprehensive community care network with multidisciplinary collaboration [46]. However, research focusing on community care networks during the COVID-19 pandemic is limited. Medical, nursing, social, and administrative personnel are expected to become exhausted during the pandemic. To respond appropriately to such circumstances, it is essential to strengthen the support systems for community care networks, including welfare commissioners. Furthermore, the evaluation of effectiveness is not necessarily limited to health outcomes, and there is a growing need for evaluation based on other values, such as strengthening the autonomy of residents and fostering social connections [47]. During the pandemic, it is important to provide resources that support relationship-building within the community. In other words, even during a pandemic, older people who are concerned about their health or lifestyle need opportunities to consult with others on a daily basis [48,49,50]. The five items extracted in this study represent information that can be managed by people who are not medical or social care experts. We speculate that sharing information that does not require advanced knowledge or skills and alerting each other to problems is versatile within a community.

This study had several limitations. First, random sampling was not performed, limiting the representativeness of the sample. As a result, the distribution of the age groups and sexes may not accurately reflect the broader population in the area. Although this study focused on a cohort of community-dwelling older people, comparisons and interpretations of the results must be made with caution, considering the inclusion criteria.

Second, due to the shorter study period, data on activities of daily living, cognitive function, and socioeconomic status were limited. Third, since information on non-responses to the baseline survey was unavailable, selection bias may have occurred. Additionally, although multiple imputations were used to address missing values, we did not address potential issues related to missing data and, therefore, could not address systematic patterns of missing responses that may cause bias. Therefore, potential sources of sampling bias, such as self-selection bias and a lack of coverage, should be examined, and the methods used to mitigate these biases, such as weighting adjustments and stratified sampling, should be discussed.

Although there are limitations to this study, there is limited knowledge regarding supporting community-dwelling older people during social distancing since the beginning of the COVID-19 epidemic. Given the limited survey items available during the COVID-19 pandemic, we believe that it is significant that we were able to suggest the characteristics of community-dwelling older people who are likely to have difficulty continuing mail surveys during social distancing. In addition, it may be useful to consider and respond to the personal factors identified in this study during the next infectious disease crisis.

## 5. Conclusions

This study underscores that if social distancing measures are reintroduced in the future, we may not be able to expect continued information exchange by mail if the following conditions apply: living alone, having comorbidities, using long-term care insurance, feeling fatigued, or experiencing a decrease in communication opportunities. Therefore, prioritizing face-to-face support is essential. In addition, even in face-to-face interactions, maintaining communication opportunities should be the key focus. This can be achieved by engaging residents who meet the basic characteristics, such as living alone, having comorbidities, and using long-term care insurance, about their recent feelings of fatigue. This approach not only benefits those who respond but also reduces the disadvantages faced by those who do not (or cannot) respond.

## Figures and Tables

**Table 1:** Group comparisons of age, sex, comorbidity, living arrangement, long-term care insurance, fall history, and total FSI scores.

	Overall	Responding Group	Non-Responding Group	t-Value	χ^2^	*p*-Value
	(n = 935)	(n = 330)	(n = 605)			
Age, mean ± standard deviation	79.09 ± 6.16	79.37 ± 5.44	78.95 ± 6.52	1.009		0.313
Sex, (male/female), n (%)	238(25.4)/697(74.6)	71(21.5)/259(78.5)	167(27.6)/438(72.4)		3.856	0.041
Living arrangement, (living together/alone), n (%)	622(66.5)/313(33.5)	256(77.6)/74(22.4)	366(60.5)/239(39.5)		27.21	<0.001
Fall history, (presence/absence), n (%)	112(12.0)/823(88.0)	38(11.5)/292(88.5)	74(12.2)/531(87.8)		0.047	0.833
Comorbidity, (presence/absence), n (%)	611(65.3)/324(34.7)	186(56.4)/144(43.6)	425(70.2)/180(29.8)		17.57	<0.001
Long-term care insurance, (use/not use), n (%)	187(20.0)/748(80.0)	54(16.4)/276(83.6)	133(22.0)/472(78.0)		3.871	0.040
FSI (score), mean ± standard deviation	1.31 ± 1.13	1.19 ± 1.08	1.38 ± 1.16	−2.529		0.014

Age, FSI score: unpaired *t*-test. Sex living, arrangement, fall history, comorbidity, and long-term care insurance: chi-square test.

**Table 2:** Group comparisons of FSI sub-items.

No.	Questions	Overall (n = 935)	Responding Group (n = 330)	Non-Responding Group (n = 605)	χ^2^	*p*-Value
Q1	Have you lost 2–3 kg or more in the past 6 months? (score: 0/1), n (%)	111(11.9)/824(88.1)	35(10.6)/295(89.4)	76(12.6)/529(87.4)	0.605	0.399
Q2	Do you think you walk slower than before? (score: 0/1), n (%)	526(56.3)/409(43.7)	176(53.3)/154(46.7)	350(57.9)/255(42.1)	1.592	0.190
Q3	Do you do physical exercise like walking at least once a week? (score: 0/1), n (%)	277(29.6)/658(70.4)	88(26.7)/242(73.3)	189(31.2)/416(68.8)	1.928	0.155
Q4	Can you recall what happened 5 min ago? (score: 0/1), n (%)	844(90.3)/91(9.7)	302(91.5)/28(8.5)	542(89.6)/63(10.4)	0.697	0.358
Q5	Have you felt tired for no reason (in the past 2 weeks)? (score: 0/1), n (%)	224(24.0)/711(76.0)	65(19.7)/265(80.3)	159(26.3)/446(73.7)	4.726	0.025

Q1–5: chi-squared test.

**Table 3:** Group comparisons for QCL sub-items.

No.	Questions	Overall	Responding Group	Non-Responding Group	t-Value	*p*-Value
		(n = 935)	(n = 330)	(n = 605)		
Q1	Amount of daily movement, mean ± standard deviation	2.47 ± 0.84	2.48 ± 0.85	2.47 ± 0.85	0.237	0.812
Q2	Leg muscle strength, mean ± standard deviation	2.31 ± 0.74	2.35 ± 0.75	2.30 ± 0.75	1.057	0.291
Q3	Meal size, mean ± standard deviation	2.80 ± 0.57	2.85 ± 0.57	2.76 ±0.58	1.725	0.085
Q4	Worry or anxiety, mean ± standard deviation	2.51 ± 0.74	2.54 ± 0.73	2.51 ± 0.76	0.624	0.532
Q5	Opportunities to talk to people, mean ± standard deviation	2.27 ± 0.86	2.18 ± 0.82	2.32 ± 0.89	−2.458	0.014

Q1–5: unpaired *t*-test.

**Table 4:** The results of the binominal logistic regression analysis.

Independent Variable	Odds Ratio	95% Confidence Interval	*p*-Value
Sex	0.84	0.67–1.18	0.328
Living arrangement (alone)	2.36	1.71–3.27	<0.001
Comorbidity (presence)	1.84	1.38–2.47	<0.001
Long-term care insurance (use)	1.69	1.15–2.49	0.007
Have you felt tired for no reason (in the past 2 weeks)? (score 1)	1.57	1.10–2.23	0.012
Opportunities to talk to people (declining trend)	1.20	1.02–1.42	0.029

Binomial logistic regression analysis was performed after the four items with significant differences were forcibly inputted and adjusted according to age and a history of falls. Hosmer–Lemeshow goodness of fit test. X-squared = 3.0447, df = 8, *p*-value = 0.931.

## Data Availability

The data supporting the findings of this study are available from the corresponding author [A.M.] upon request.

## References

[1] Ministry of Internal Affairs and Communications Statistics Bureau Home Page: Statistical Topics No. 142 Japan’s Older Adults Seen from Statistics—In Honor of “Respect for the Aged Day”. https://www.stat.go.jp/data/topics/pdf/topics142.pdf.

[2] Ministry of Health, Labor and Welfare Healthy Life Expectancy in 2022 (Reiwa 4). https://www.mhlw.go.jp/content/10904750/001363069.pdf.

[3] Lewis S., Ewald L., Duber H.C., Mokdad A.H., Gakidou E. (2024). Determinants of Unmet Healthcare Needs During the Final Stage of the COVID-19 Pandemic: Insights From a 21-Country Online Survey. Int. J. Public Health.

[4] Na L. (2022). Characteristics of community-dwelling older individuals who delayed care during the COVID-19 pandemic. Arch. Gerontol. Geriatr..

[5] Sands L.P., Albert S.M., Suitor J.J. (2020). Understanding and addressing older adults’ needs during COVID-19. Innov. Aging.

[6] Steinman M.A., Perry L., Perissinotto C.M. (2020). Meeting the care needs of older adults isolated at home during the COVID-19 pandemic. JAMA Intern. Med..

[7] Li W., Frydman J.L., Li Y., Liu B. (2022). Characterizing delayed care among US older adults by self-rated health during the COVID-19 pandemic. Prev. Med..

[8] Jones A.N., Power M.C. (2023). Pre-pandemic factors associated with delayed health care among US older adults during the COVID-19 pandemic. J. Med. Access.

[9] Zhong S., Huisingh-Scheetz M., Huang E.S. (2022). Delayed medical care and its perceived health impact among US older adults during the COVID-19 pandemic. J. Am. Geriatr. Soc..

[10] Yamada M., Kimura Y., Ishiyama D., Otobe Y., Suzuki M., Koyama S., Kikuchi T., Kusumi H., Arai H. (2021). The influence of the COVID-19 pandemic on physical activity and new incidence of frailty among initially non-frail older adults in Japan: A follow-up online survey. J. Nutr. Health Aging.

[11] Ataka T., Kimura N., Eguchi A., Matsubara E. (2022). Changes in objectively measured lifestyle factors during the COVID-19 pandemic in community-dwelling older adults. BMC Geriatr..

[12] van Der Klei V.M.G.T.H., Moens I.S., Simons T., den Elzen W.P.J., Mooijaart S.P., Gussekloo J., Trompet S., Drewes Y.M., Drewes Y.M. (2024). The impact of the COVID-19 pandemic on Positive Health among older adults in relation to the complexity of health problems. J. Am. Geriatr. Soc..

[13] Alonzi S., La Torre A., Silverstein M.W. (2020). The psychological impact of preexisting mental and physical health conditions during the COVID-19 pandemic. Psychol. Trauma.

[14] Mitra A.R., Fergusson N.A., Lloyd-Smith E., Wormsbecker A., Foster D., Karpov A., Crowe S., Haljan G., Chittock D.R., Kanji H.D. (2020). Baseline characteristics and outcomes of patients with COVID-19 admitted to intensive care units in Vancouver, Canada: A case series. CMAJ.

[15] Wong S.Y.S., Zhang D., Sit R.W.S., Yip B.H.K., Chung R.Y.N., Wong C.K.M., Chan D.C.C., Sun W., Kwok K.O., Mercer S.W. (2020). Impact of COVID-19 on loneliness, mental health, and health service utilisation: A prospective cohort study of older adults with multimorbidity in primary care. Br. J. Gen. Pract..

[16] Vannini P., Gagliardi G.P., Kuppe M., Dossett M.L., Donovan N.J., Gatchel J.R., Quiroz Y.T., Premnath P.Y., Amariglio R., Sperling R.A. (2021). Stress, resilience, and coping strategies in a sample of community-dwelling older adults during COVID-19. J. Psychiatr. Res..

[17] Whitehead B.R. (2021). COVID-19 as a stressor: Pandemic expectations, perceived stress, and negative affect in older adults. J. Gerontol. B Psychol. Sci. Soc. Sci..

[18] Koizumi S., Ohta A., Kamei M. (2024). Homebound older adults who live independently in rural Japan: Prevalence and contributing factors during the COVID-19 pandemic. Prev. Med. Rep..

[19] Shinohara T., Saida K., Tanaka S., Murayama A. (2020). Rapid response: Impact of the COVID-19 pandemic on frailty in the elderly citizen; corona-frailty. BMJ.

[20] Shinohara T., Saida K., Tanaka S., Murayama A. (2020). Do lifestyle measures to counter COVID-19 affect frailty rates in elderly community dwelling? Protocol for cross-sectional and cohort study. BMJ Open.

[21] Tanaka S., Saida K., Murayama A., Higuchi D., Shinohara T. (2023). Associated factors of new subjective cognitive decline complaints after a 6-month period among community-dwelling older adults during the COVID-19 pandemic in Japan. Psychogeriatrics.

[22] Tanaka S., Murayama A., Higuchi D., Saida K., Shinohara T. (2023). Relationship between consistent subjective cognitive decline and occurrence of falls six months later. Arch. Gerontol. Geriatr..

[23] Murayama A., Higuchi D., Saida K., Tanaka S., Shinohara T. (2024). Fall risk prediction for community-dwelling older adults: Analysis of assessment scale and evaluation items without actual measurement. Int. J. Environ. Res. Public Health.

[24] Gunma Prefectural Office: Temporary Press Conference on Requests for Priority Measures Such as Prevention of Spread (May 12). https://www.pref.gunma.jp/site/chiji/22640.html.

[25] Ministry of Health, Labor and Welfare Response to the Novel Coronavirus Infection After Shift to Category 5 Infectious Disease. https://www.mhlw.go.jp/stf/corona5rui.html.

[26] Yamada M., Arai H. (2015). Predictive value of frailty scores for healthy life expectancy in community-dwelling older Japanese adults. J. Am. Med. Dir. Assoc..

[27] Shinohara T., Saida K., Tanaka S., Murayama A. (2021). Actual frailty conditions and lifestyle changes in community-dwelling older adults affected by coronavirus disease 2019 countermeasures in Japan: A cross-sectional study. Sage Open Nurs..

[28] Watanabe D., Yoshida T., Watanabe Y., Yamada Y., Miyachi M., Kimura M. (2022). Validation of the Kihon Checklist and the frailty screening index for frailty defined by the phenotype model in older Japanese adults. BMC Geriatr..

[29] Satake S., Arai H. (2020). The revised Japanese version of the Cardiovascular Health Study criteria (revised J-CHS criteria). Geriatr. Gerontol. Int..

[30] Fried L.P., Tangen C.M., Walston J., Newman A.B., Hirsch C., Gottdiener J., Seeman T., Tracy R., Kop W.J., Burke G. (2001). Frailty in older adults: Evidence for a phenotype. J. Gerontol. A Biol. Sci. Med. Sci..

[31] O’Connell M.L., Coppinger T., McCarthy A.L. (2020). The role of nutrition and physical activity in frailty: A review. Clin. Nutr. ESPEN.

[32] Shimada H., Makizako H., Lee S., Doi T., Lee S., Tsutsumimoto K., Harada K., Hotta R., Bae S., Nakakubo S. (2016). Impact of cognitive frailty on daily activities in older persons. J. Nutr. Health Aging.

[33] Makizako H., Shimada H., Doi T., Yoshida D., Anan Y., Tsutsumimoto K., Uemura K., Liu-Ambrose T., Park H., Lee S. (2015). Physical frailty predicts incident depressive symptoms in elderly people: Prospective findings from the Obu Study of Health Promotion for the Elderly. J. Am. Med. Dir. Assoc..

[34] Shinohara T., Saida K., Tanaka S., Murayama A. (2021). Association between frailty and changes in lifestyle and physical or psychological conditions among older adults affected by the coronavirus disease 2019 countermeasures in Japan. Geriatr. Gerontol. Int..

[35] Tierney N., Cook D., McBain M., Fay C., O’Hara-Wild M., Hester J., Smith L., Heiss A. Package “naniar”. https://cran.r-project.org/web/packages/naniar/naniar.pdf.

[36] Van Buuren S., Groothuis-Oudshoorn K. (2011). mice: Multivariate imputation by chained equations in R. J. Stat. Softw..

[37] van Buuren S., Groothuis-Oudshoorn K., Vink G., Schouten R., Robitzsch A., Rockenschaub P., Doove L., Jolani S., Moreno-Betancur M. Package “mice”. https://cran.r-project.org/web/packages/mice/mice.pdf.

[38] White I.R., Royston P., Wood A.M. (2011). Multiple imputation using chained equations: Issues and guidance for practice. Stat. Med..

[39] Harrell F.E., Lee K.L., Califf R.M., Pryor D.B., Rosati R.A. (1984). Regression modelling strategies for improved prognostic prediction. Stat. Med..

[40] Kanda Y. (2013). Investigation of the freely available easy-to-use software “EZR” for medical statistics. Bone Marrow Transplant..

[41] Maruta J., Kurozumi H., Uchida K., Akada S., Inoue K. (2024). Longitudinal changes in anxiety and depression and their ameliorating lifestyle factors among community-dwelling older adults during the COVID-19 pandemic. Arch. Gerontol. Geriatr. Plus.

[42] Murayama A., Higuchi D., Saida K., Tanaka S., Shinohara T. (2024). Risk factors for falls in community-dwelling older adults during the novel coronavirus pandemic in Japan: A prospective cohort study. Int. J. Environ. Res. Public Health.

[43] World Health Organization WHO to Identify Pathogens That Could Cause Future Outbreaks and Pandemics. https://www.who.int/news/item/21-11-2022-who-to-identify-pathogens-that-could-cause-future-outbreaks-and-pandemics.

[44] Chen Z., Cong Z. (2023). Age differences in experiencing negative impacts of the COVID-19 pandemic: A latent class analysis. Int. J. Disaster Risk Reduct..

[45] Sugaya N., Yamamoto T., Uchiumi C. (2024). A 2-year longitudinal study examining the change in psychosocial factors under the COVID-19 pandemic in Japan. Sci. Data.

[46] Katori T. (2024). Japan’s healthcare delivery system: From its historical evolution to the challenges of a super-aged society. Glob. Health Med..

[47] Weber P., Birkholz L., Straub R., Kohler S., Helsper N., Dippon L., Pfeifer K., Rütten A., Semrau J. (2024). The limitations and potentials of evaluating economic aspects of community-based health promotion: A critical review. Appl. Health Econ. Health Policy.

[48] Su Y., Hamatani M., Yuki M., Ogawa N., Kawahara K. (2024). Frailty and social isolation before and during the coronavirus disease 2019 pandemic among older adults: A path analysis. J. Adv. Nurs..

[49] Hamatani M., Su Y., Yuki M., Ogawa N., Kawahara K. (2024). Depressive symptoms associated with infection prevention measures and daily lifestyle habit characteristics among Japanese community-dwelling older adults during the COVID-19 pandemic. Jpn. J. Nurs. Sci..

[50] Tokumitsu K., Sugawara N., Tabuchi T., Yasui-Furukori N. (2024). Real-world predictors of changes in fear of COVID-19 in the Japanese general population: A large-scale internet-based cohort study with 20,712 participants. BMC Psychiatry.

